# Classification of chronic radiation-induced ulcers in the chest wall after surgery in breast cancers

**DOI:** 10.1186/s13014-017-0876-y

**Published:** 2017-08-15

**Authors:** Xiao Ma, Zengqiang Jin, Guojun Li, Wenfeng Yang

**Affiliations:** 1 Key Laboratory of Carcinogenesis and Translational Research (Ministry of Education), Department of Head and Neck, Perking University Cancer Hospital and Institute, Beijing, 100142 China; 2 Department of Plastic Surgery, The Affiliated Hospital of Academy of Military Medical Sciences, Beijing, 100043 China; 3 Department of Head and Neck Surgery, Unit 1445, The University of Texas MD Anderson Cancer Center, 1515 Holcombe Boulevard, Houston, TX 77030 USA

**Keywords:** Chest wall, Radiation-induced ulcer, Myocutaneous flap, Filleted flap, Classification, Breast cancer

## Abstract

**Background and purpose:**

To explore the methods of clinical classification in chronic radiation-induced ulcers in the chest wall (CRUCWs).

**Materials and methods:**

A total of 64 patients with CRUCWs were treated. We divided the cases into 3 types (mild, moderate, or severe) according to their clinical manifestations. Conservative treatments, axial-pattern myocutaneous or local flaps, or filleted flaps were applied correspondingly.

**Results:**

The cases were divided as follows: mild (*n* = 11), moderate (*n* = 45), and severe (*n* = 8). Eight cases were cured by conservative surgical therapy. One case had a recurrence 6 months after conservative therapy and was cured by a latissimus dorsi myocutaneous flap. The transferred flaps all survived, including 26 transverse rectus abdominis myocutaneous flaps, 8 longitudinal rectus abdominis myocutaneous flaps, 6 latissimus dorsi myocutaneous flaps, 3 contralateral breast flaps, 5 lateral thoracic rotation flaps, and 7 filleted flaps. In 2 transverse rectus abdominis myocutaneous flaps and 2 latissimus dorsi myocutaneous flaps, distal necrosis appeared in small areas. The resulting wounds were salvaged with skin graft and full healing was achieved.

**Conclusion:**

CRUCWs can be divided into three types. Surgical methods should vary with distinguished classifications. The effective classification of CRUCWs has definite instructive significance on the selection of surgical approaches.

## Introduction

Radiation therapy (RT) is a double-edged sword for breast cancer patients. On the one hand, RT reduces the risk of local recurrence and increases survival rates; On the other hand, it may lead to severe adverse effects on normal tissue such as radiation-induced ulcers and osteoradionecrosis [1, 2]. Radiation-induced lesions may appear up to 10 years after irradiation and are initiated by damage to the vascular endothelial microvessels [3]. This damage eventually causes non-healing ulcers and soft tissue necrosis. The decreased angiogenesis and persistently high concentration of matrix metalloproteinase indicate a molecular environment in chronic wounds that is hostile to cell replication after injury [4–6]. Radiation-induced ulcers have secondary, progressive, and irreversible characteristics. Once chronic radiation-induced ulcers in the chest wall (CRUCWs) are formed, they are difficult to heal because of the reduced blood supply, fibrosis, and impaired cellular repair potential associated with radiation therapy [7, 8]. The treatment of CRUCWs is further complicated because they are often combined with radiation osteomyelitis, radiation pneumonitis, brachial plexus injury, and other comorbidities [7, 9].

The clinical manifestations of CRUCWs are diverse, and ulcer size, location, depth, and basal conditions can vary widely. These factors determine which of several different treatment and/or reconstruction methods are used to treat CRUCWs in clinical practice, but the selection of treatment for CRUCW is not optimized. So under these considerations, the purpose of this study was to further refine the diagnosis of CRUCW by accurately typing CRUCW severity according to its clinical manifestations to guide clinical treatment.

We divided 64 cases of CRUCW into 3 types according to clinical manifestations. Conservative treatments, rotation of axial-pattern myocutaneous and local flaps, or filleted flaps were applied correspondingly.

## Materials and methods

### Patient selection

We searched the patient records at our institution and identified 64 patients who had been diagnosed with CRUCW from January 2001 to December 2012. CRUCW was defined as an ulcer or ulcers in the irradiated region of the chest wall after mastectomy. Biopsies were performed prior to treatment planning to eliminate the possibility of cancer recurrence or radiation-induced sarcoma. If a CRUCW was confirmed, surgical management was required. This study was approved by the local Research Ethics Board, and informed consent was obtained from all patients. Patient follow-up period was 2.6 years (range 1–4). The ulcer repairing situation was assessed by professionals.

### Data collection

We obtained demographic, clinical, and treatment information as well as follow up from the patients’ records. The following clinical and CRUCW characteristics were recorded: ulcer size, position, shape, and depth; granulation tissue of the ulcer base, surrounding skin color and texture, morphology and function of the upper limbs, and presence of associated bone necrosis or heart and lung disease.

### Classification for CRUCW

Prior to classification for CRUCW, all patients underwent computed tomography, magnetic resonance imaging, X-ray imaging, ultrasonography, and electromyography. Emphasis was placed on whether the CRUCW was associated with radiation pneumonitis, collarbone and/or rib osteonecrosis, and/or radioactive brachial plexus injury.

The LENT-SOMA (late effects of normal tissue–subjective, objective, management, and analytic criteria) of breast scale represents a good tool to score toxicity after RT. While surgical treatment is chosen for CRUCW, this scale lacks clear guidance. Thus, we divided the CRUCWs into three types based on its clinical manifestations: 1) mild type: the ulcer area was less than 10cm^2^; the depth reached the subcutaneous layer; and there was a relatively healthy granulation tissue in ulcer base without any upper limb movement disorder; 2) moderate type: the ulcer area was larger than 10cm^2^ and was accompanied by partial bone exposure in ulcer base without health granulation tissue, and partially upper limb sensory as well as motor disorders; and 3) severe type: the ulcer area was larger than 100cm^2^, and was accompanied by obvious collarbone and/or rib osteonecrosis in ulcer base, as well as complete loss of upper limb function (Table 1).

**Table 1:** CRUCW classification

Characteristics	CRUCWs(*N* = 64)		
	Mild (*N* = 11)	Moderate(*N* = 45)	Severe(*N* = 8)
Area (cm^2^)			
≤ 10	11	16	7
> 100	0	29	1
Depth			
subcutaneous	11	0	0
muscle layer	0	22	0
pleural layer or deeper	0	23	8
Skin fibrosis			
mild	11	15	0
severe	0	30	8
Radiation pneumonitis			
yes	0	11	8
no	11	34	0
Ribs and/or collarbone ORN			
yes	0	12	8
no	11	33	0
Lymphedema, arm			
≤ 6 cm	11	34	0
> 6 cm	0	11	8
Brachial neuropathy			
yes	0	4	8
no	11	41	0

*Abbreviation*: *CRUCWs* Chronic radiation-induced ulcers in chest wall, *ORN* osteonecrosis

### Treatment and evaluation

The patients were treated with one of the following three surgical methods: (1) Conservative therapy: simple debridement was performed until healthier basal granulation tissue appeared. Then the wounds were covered with silver sulfadiazine dressings or Vaseline gauze. The dressing could be changed every 2–3 days according to the rate of drainage. After 2007, a vacuum sealing drainage (VSD) apparatus was adopted for processing wounds. The VSD system could be changed for once or twice during the wound-healing process. Stamp skin grafting was used to cover the wound when the basal granulation tissue began growing well. (2) Axial-pattern flaps and local flaps: once the ulcer became larger, basal conditions worsened, more severe fibrosis of the surrounding skin emerged, or bone tissue exposure and/or necrosis occurred, a vascularized flap became necessary. After debridement, the wound was repaired using one of a variety of flaps, the most popular of which were the rectus abdominis myocutaneous flaps (RAM), latissimus dorsi myocutaneous flaps (LDM), and contralateral breast and lateral thoracic rotation flaps. (3) Filleted flaps: when the CRUCW was accompanied by a severely function-impaired upper limb due to irreversible and chronic progressive brachial plexus injury and lymphedema, the ulcers became huge wounds that were hard to repair with conventional methods, left necrotic ribs and collarbone exposed, and caused the patients to suffer from refractory and excruciating pain. After the patient’s opinion was carefully solicited, filleted flaps from the dysfunctional ipsilateral upper limbs were used to repair the wounds. All the patients’ wounds were cured within 1–4 years after follow up.

### Statistical analysis

The frequency distributions of patient characteristics, including age, duration of disease, the total doses of radiation, CRUCWs classifications and reconstruction methods were calculated and summarized. The SPSS 19.0 statistical software package is used to process data. The Pearson Chi-square test and Fisher’s exact test were used to compare differences between categorical variables as appropriate. All tests of statistical significance were two-tailed with a *p* value of 0.05 or less considered significant.

## Results

### Patient characteristics

The 64 patients were exclusively female, aged 22 to 75 years with a mean age of 54 years (Table 2). Ulcers emerged from 3 to 24 years after radiotherapy, with an average duration of 11 years. The severity of CRUCWs had an obvious dose-effect relationship with the radiation dose (*p* < 0.001). Furthermore, the CRUCW type was associated with the age of onset and course of disease. In older patients and those with longer course of CRUCW, the degree of radiation damage was more severe (*p* < 0.001; *p* = 0.011).

**Table 2:** Baseline characteristics

Factors	CRUCWs (*n* = 64)			*p*
	Mild (*n* = 11)	Moderate (*n* = 45)	Severe (*n* = 8)	
Ages (years)				<0.001
Median (range)	39.8 (22–61)	47.3 (34–68)	64.5 (57–75)	
< 60	9	39	1	
> 60	2	6	7	
Duration of disease (years)				0.011
≤ 10	9	22	1	
> 10	2	23	7	
Total amount of radiation (cGy)				<0.001
≤ 5000	10	19	0	
> 5000	1	26	8	

*Abbreviation*: *CRUCWs* Chronic radiation-induced ulcers in chest wall

### Classification

The 64 CRUCWs were divided into mild (*n* = 11), moderate (*n* = 45), and severe (*n* = 8) types.

### Therapies and complications

**Fig. 1:**
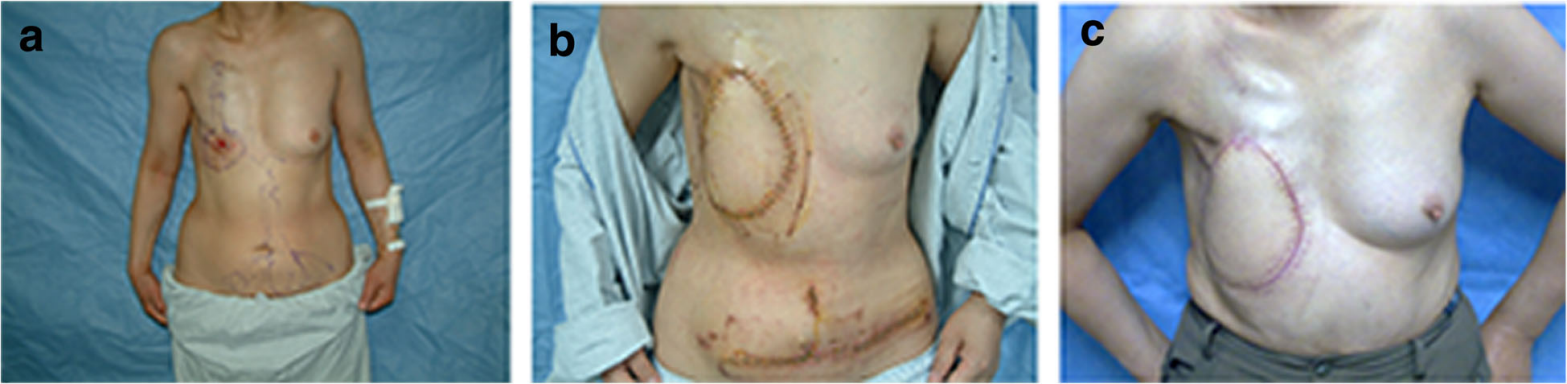
**a** A 34-year-old patient underwent 50-Gy radiation therapy due to breast cancer after tumor ablation. A mild CRUCW was observed. The patient had a high aesthetic requirement. **b** TRAM flap was designed to repair the wound. **c** One year after the surgery the TRAM flap reconstruction had healed completely

**Fig. 2:**
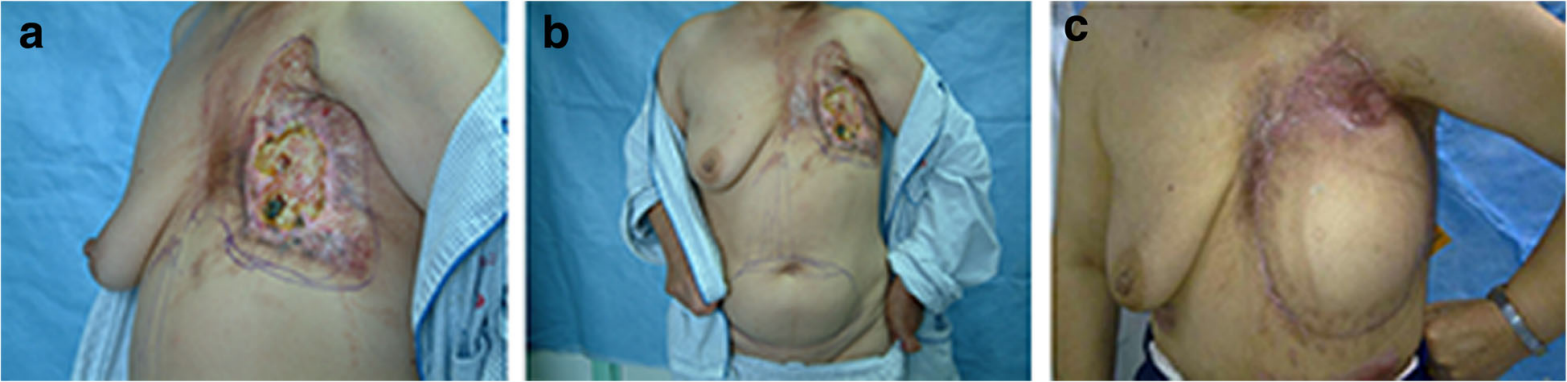
**a** A 56-year-old patient underwent 65-Gy radiation treatment due to breast cancer after tumor ablation. A moderate CRUCW was observed with 3–4 necrotic ribs in the center of the CRUCW. **b** A TRAM flap was designed to repair the wound. **c** Two years after the surgery the radiation ulcer had healed completely

**Fig. 3:**
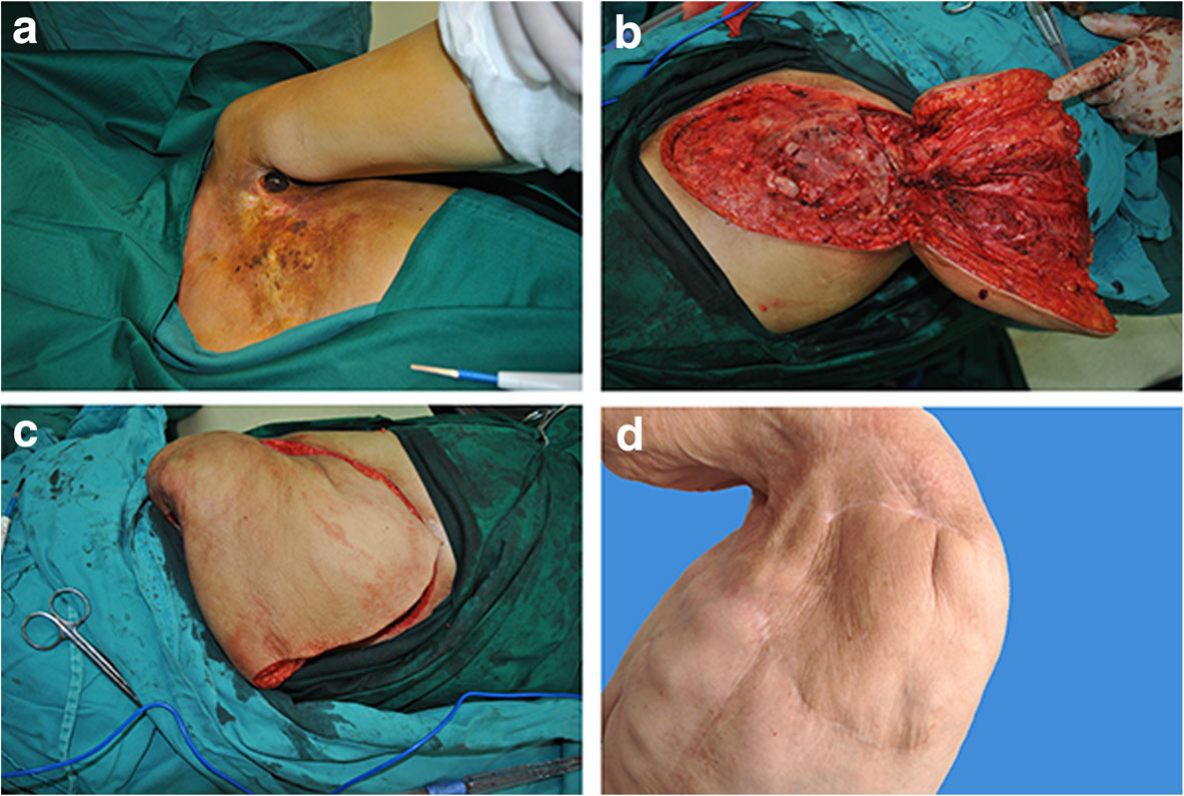
**a** A 76-year-old patient underwent 80-Gy radiation treatment due to breast cancer after tumor ablation. A severe CRUCW was observed. **b** Intraoperative examination showed a necrotic partial clavicle and 2–4 ribs that were ablated with the parietal pleura. A filleted flap was made to repair the wounds using the dysfunctional ipsilateral upper limbs. **c** The filleted flap fitted the huge 35 cm X 25 cm wound. **d** Two years after the surgery, the radiation ulcer had healed completely

Among the mild CRUCWs, 8 cases were cured by conservative surgical treatment. In 2 cases in young patients, the CRUCW was repaired by a transverse rectus abdominis myocutaneous flap (TRAM) with an acceptable aesthetic outcome (Figure 1). One case had a recurrence 6 months after conservative surgical treatment and was cured by a LDM flap. Among the 45 medium CRUCW cases, 23 were reconstructed with TRAM flaps (Figure 2), 10 with LRAM flaps, 6 with LDM flaps, 3 with CLB flaps, and 5 with LTR flaps. Two of the TRAM flaps and 2 of the LDM flaps developed small areas of distal necrosis. The resulting wounds were salvaged with skin grafts, and full healing was achieved. Eight severe CRUCWs were treated by 7 filleted flaps (Figure 3) and 1 TRAM flap. One filleted flap developed a postoperative infection due to incomplete clavicle debridement and formed a chronic fistula, resulting in delayed healing due to anti-inflammatory treatment and another debridement. After 1 to 4 years’ follow-up, all the wounds were cured (Table 3).

**Table 3:** Surgical reconstruction of CRUCWs

Reconstruction	CRUCWs(*n* = 64)			Complications
	Mild (*n* = 11)	Moderate(*n* = 45)	Severe(*n* = 8)	
Conservation	9	—	—	one recurred, cured by LDM flap
Axial-pattern/local flaps				
TRAM	2	23	1	two distal partial flap necrosis, then cured by skin grafting
LRAM	—	8	—	—
LDM	—	6	—	two small area of focal necrosis, then cured by skin grafting
CLB	—	3	—	—
LTR	—	5	—	—
Filleted flap	—	—	7	one wound infection, delayed healing after secondary staged debridement

*Abbreviation*: *TRAM* transverse rectus abdominis myocutaneous flap, *LRAM* lonti-tudinal rectus abdominis myocutaneous flap, *LDM* latissimus dorsi musculo-cutaneous flap, *CLB* contralateral breast flap, *LTR* lateral thoracic rotation flap

## Discussion

Based on the clinical features and severity of complications, we divided CRUCWs into 3 types: mild, moderate, and severe. This classification may help and guide surgeons for appropriate diagnosis and treatment for CRUCW. Clinically, we found that, except for mild CRUCWs with healthy basal granulation tissue, which could be cured by wound dressing and conservative treatment, most of the ulcers in our study had fibrosis in and around the base and scar tissue with a poor blood supply, which required skin or muscle flaps with a good blood supply to cover the wound.

In our study, we demonstrated that the severity of CRUCWs has an obvious dose-effect relationship with the radiation dose. Furthermore, CRUCW severity increased with the age of onset and latency. Previous studies [10–12] have shown that the irradiation method, the presence of chemotherapy, the patient’s own tolerance to radiation, and other factors may influence the emergence of radiation injury. Further study is needed to more precisely delineate the effects of these and other factors, alone and in combination, on radiation injury.

Our CRUCW classification method is a useful supplement to the LENT-SOMA breast module. The LENT-SOMA scoring system, first published in 1995, has become widely used within the past few years [13]. The LENT-SOMA system is a good tool for scoring radiation toxicity; however, many of its recommendations have been designed for use in clinical trials of new treatment methods and little attention has been given to the rather different needs of routine practice [14]. The purpose of this article is to guide the surgical treatment for CRUCW by further refinement of diagnosis of it. Therefore, the mild type could be completely cured by conservative therapy; debridement followed by immediate coverage with axial-pattern myocutaneous or local flaps might be the best choice for treatment of the moderate type; and the filleted flaps should be considered for severe type.

One severe CRUCW case in our study had a long-term fistula after the first operation due to incomplete debridement of the necrotic collarbone and needed another debridement to remove necrotic tissue and heal the wound. Surgical debridement should follow the principle of great breadth and depth control. Great breadth means the area of the surgical resection should be large enough to not only include the ulcer but also any apparent radiological changes to the skin tissue around the ulcer. This ensures that the transferred muscle flap will be attached to relatively healthy skin with an adequate blood supply. At the same time, any obvious radiation-induced osteomyelitis of the sternum, ribs, or clavicle should be excised. Bone lesions appear as gray-black with a soft or waxy texture, and affected bone marrow cavities have no bleeding points. Depth control means the debridement depth should be controlled outside the pleural cavity. Generally, the pleura have good tolerance to radiotherapy, and it is likely to bring infection into the thoracic cavity if the pleural cavity is opened. Thus, we use a blade to carefully scrape the fibrotic pleura until a little bleeding of the base can be seen. Then, the transferred muscle flap with a rich blood supply can nourish the deep tissue and achieve a clear biological benefit.

Compared with skin flaps, muscle flaps have a good blood supply, an abundant amount of tissue, and transferred muscle that can be used to fill deep defects. Therefore, for more complex CRUCWs, muscle flaps are our clinical choice. The RAM flap, one of the oldest myocutaneous flaps, is used for thoracic wall reconstruction and breast reconstruction [15, 16]. It is one of the most commonly used flaps in reconstructive surgery because of its constant pedicle, rich amount of tissue, and easy manipulation. In our study, the case with the greatest defect area (500 cm^2^) and the highest defect location (reaching the collarbone plane) was repaired by a RAM flap. LDM flaps share similar characteristics with RAM flaps but contain a smaller amount of muscle tissue. For smaller tissue defects, lateral chest flaps or contralateral breast flaps may also be employed.

Filleted flaps require sacrificing an upper limb and thus are a heavy psychological and appearance blow for the patient. There are strict indications for the use of filleted flaps: huge CRUCWs involving multiple anatomical areas that are hard to repair; pain that seriously affects the patient’s quality of life, and/or CRUCWs that threaten the patient’s life. Also, the upper limb must be in disuse due to severe brachial plexus injury or severe lymphedema or have little significance for the patient and the likelihood of success using conventional repair methods must be extremely low [17, 18].

Free flaps and perforator flaps may also be useful for soft tissue coverage options. Some studies have reported a superior arterial flow, a larger angiosome and less minor and major complications for the inferior epigastric artery/free TRAM-flap as compared to the superior epigastric artery/pedicled TRAM flap. Therefore, at least for the type 1 and possibly 2 CRUCW free flap surgery, it might be the optional method for choice wherever recipient vessels (internal mammary or thoracodorsal artery) are still present. For those patients lacking sufficient recipient vessels, arteriovenous loops (as single- or two-stage procedures) in combination have been shown to be a safer option with less minor complications, as compared to large and/or multiple pedicled flaps [19, 20]. Since this is a relatively new approach and has not yet been established for most of the plastic surgery units, currently pedicled flaps might still pose a good (and only) option under these circumstances (complete lack of recipient vessels for free flap transplantation).

In considering the clinical relevance and importance of our findings, several limitations and nuances should be noted. The sample size of our study was not large enough and the design lack of prospective. One mild case first cured by a conservative operation recurred 6 months later at the same site. This situation requires close attention. That also showed the complexity and diversity of CRUCW. The rationality of such classification needs a longer follow-up of patients and further study.

## Conclusion

In summary, according to its different clinical manifestations, CRUCW can be divided into 3 types, which should determine the surgical treatment method used. The effective typing of CRUCWs has definite instructive significance on the selection of surgical approaches.

## Abbreviations

CLB: Contralateral breast flap
CRUCW: Chronic radiation-induced ulcers in the chest wall
LDM: Latissimus dorsi myocutaneous flaps
LENT-SOMA: Late effects of normal tissue–subjective, objective, management, and analytic criteria
LTR: Lateral thoracic rotation flap
RAM: Rectus abdominis myocutaneous flaps
TRAM: Transverse rectus abdominis myocutaneous flap
